# Modeling of Hydrogen Diffusion in Inhomogeneous Steel Welded Joints

**DOI:** 10.3390/ma15217686

**Published:** 2022-11-01

**Authors:** Andrei I. Rudskoi, Victor A. Karkhin, Egor B. Starobinskii, Sergey G. Parshin

**Affiliations:** Institute of Mechanical Engineering, Materials and Transport, Higher School of Physics and Materials Technologies, Peter the Great St. Petersburg Polytechnic University, St. Petersburg 195251, Russia

**Keywords:** hydrogen, diffusion, weld inhomogeneity, diffusion coefficient, solubility, steel, modeling

## Abstract

Hydrogen is a main factor in cold cracking or hydrogen-induced cracking. The most crack susceptible region of a steel welded joint is the heat affected zone (HAZ). The formulation and functional-analytical solution of the one-dimensional problem of hydrogen diffusion in an inhomogeneous butt-welded joint considering weld and joint dimensions and initial hydrogen distribution as well as hydrogen diffusion coefficients and solubilities are presented. It is shown that the peak hydrogen concentration in the HAZ of inhomogeneous joints varies in direct proportion to the initial hydrogen concentration in the weld metal. It is inversely proportional to the ratio of hydrogen solubilities in the weld metal and the HAZ metal and is nonlinear in the diffusion coefficient ratio of these metals. The peak hydrogen concentration in the HAZ can exceed 170% of the average initial concentration in the joint if martensitic steel is welded using low-carbon low-alloy welding consumables. The utilization of austenitic consumables leads to a dramatic reduction in the hydrogen concentration in the HAZ in comparison with the non-austenitic consumables. No direct relationship was found between the hydrogen concentration in the HAZ and the hydrogen evolution from the joint surface.

## 1. Introduction

It is well-known that the cold cracking of steel weldments is promoted by higher strength microstructure (such as martensite), higher tensile stresses (as those imposed in welding in restrained heavy sections), and higher levels of diffusible hydrogen. Cold cracking is also called hydrogen-induced or hydrogen-assisted cracking. Hydrogen cracking may occur in both the weld metal (WM) and the heat affected zone (HAZ), although it is most prevalent in the HAZ due to the combination of the microstructure and tensile restraint that exists in the region of the weld [1]. To assess the probability of cold cracking based on strength criteria, information on the local hydrogen concentration in crack susceptible regions is required.

There are several reliable procedures for the determination of hydrogen content in the weld and deposited metal [2]. Measurement of a local concentration of hydrogen, however, is extremely difficult due to the high mobility of hydrogen atoms in a crystalline lattice. Because of this, calculation methods are widely used in the assessment of hydrogen diffusion from the WM to the HAZ, and reasonably comprehensive physical-mathematical models for hydrogen diffusion have been developed [2,3,4,5,6,7,8]. To be utilized, such models require the application of numerical methods and in common with all physical experiments, they suffer from the drawback that the results are often difficult to generalize, and it is thus difficult to find a general rule. Functional analysis methods allow the solution of the problem to be presented as a formula, but they require considerable simplification of the physical model [6,9,10,11].

The aim of the study was to develop an approximate analytical model for hydrogen diffusion with consideration for weld and joint dimensions, different initial hydrogen concentrations in the WM and the base metal (BM), and inhomogeneity in the microstructure (diffusion coefficients and hydrogen solubilities) of the WM and BM. The model is demonstrated with different combinations of the WM and BM.

## 2. Statement of the Hydrogen Diffusion Problem

**Figure 1:**
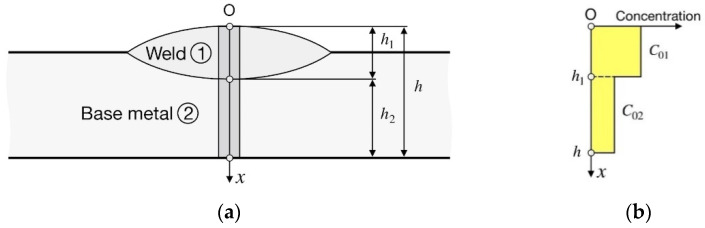
Schematic of a bead-on-plate weld: (**a**) coordinate system; (**b**) initial distribution of hydrogen concentration.

Let us make the following assumptions:

A welded joint consists of two materials (WM and BM) with different diffusion coefficients, *D*_1_ and *D*_2_, and solubilities, *S*_1_ and *S*_2_, with a stationary composite interface (subscript 1 refers to the WM and subscript 2 to the BM) (Figure 1a).

Properties *D*_1_, *D*_2_ and *S*_1_/*S*_2_ are constant at any moment.

The initial hydrogen concentration is constant in the WM (*C*_01_ = const) and in the BM (*C*_02_ = const) (Figure 1b).

Surface concentrations of hydrogen are zero because of very low hydrogen partial pressure in the air.

There are no hydrogen traps or other internal hydrogen sources and sinks.

Hydrogen flow is one-dimensional and directed along the *x*-axis and normally to the welding line (Figure 1a). This is more valid; the wider is the weld relative to the depth of penetration and the plate thickness.

The assumptions made allowed for the problem to be formulated in the following way.

(1) Mass transfer equation (Fick’s law):
  (1) $$\frac{\partial C_{1}\left(x,t\right)}{\partial t}=D_{1}\frac{\partial^{2}C_{1}\left(x,t\right)}{\partial x^{2}},\;t>0,\;0<x<h_{1}$$
  (2) $$\frac{\partial C_{2}\left(x,t\right)}{\partial t}=D_{2}\frac{\partial^{2}C_{2}\left(x,t\right)}{\partial x^{2}},\;t>0,\;h_{1}<x<h_{2},$$
  where t is the time.
(2) Initial conditions (t=0, Figure 1b):
  (3) $$C_{1}\left(x,0\right)=C_{01},$$
  (4) $$C_{2}\left(x,0\right)=C_{02}.$$
(3) Boundary conditions (Figure 1a):
  (5) $$C_{1}\left(0,t\right)=0,$$
  (6) $$C_{2}\left(h,t\right)=0.$$

Flux is continuous over the surface of separation of the two media and the partition coefficient *k*_0_ is constant, according to Nernst’s law:

(7) $$D_{1}\frac{\partial C_{1}\left(h_{1},t\right)}{\partial x}=D_{2}\frac{\partial C_{2}\left(h_{1},t\right)}{\partial x},$$

(8) $$\frac{C_{1}\left(h_{1},t\right)}{C_{2}\left(h_{1},t\right)}=\frac{S_{1}}{S_{2}}=k_{0}.$$

## 3. Solution of the Hydrogen Diffusion Problem 

The formulated linear boundary-value problem (1)–(8) can be solved by integral transformation [12]:

(9) $$C_{1}\left(x,t\right)=k_{0}\overset{\infty }{\underset{n=1}{\sum }}\left[\left(C_{01}A_{n}+C_{02}B_{n}\right)\sin\left(\beta_{n}h_{2}\right)\sin\left(k\beta_{n}x\right)\exp\left(-D_{2}\beta_{n}^{2}t\right)\right],$$

(10) $$C_{2}\left(x,t\right)=\overset{\infty }{\underset{n=1}{\sum }}\left[\left(C_{01}A_{n}+C_{02}B_{n}\right)\sin\left(k\beta_{n}h_{1}\right)\sin\left(\beta_{n}\left(h-x\right)\right)\exp\left(-D_{2}\beta_{n}^{2}t\right)\right],$$

where:

$$A_{n}=\frac{2\sin\left(\beta_{n}h_{2}\right)}{k\beta_{n}}\frac{1-\cos\left(k\beta_{n}h_{1}\right)}{h_{2}\sin^{2}\left(k\beta_{n}h_{1}\right)+k_{0}h_{1}\sin^{2}\left(\beta_{n}h_{2}\right)},$$

$$B_{n}=\frac{2\sin\left(k\beta_{n}h_{1}\right)}{\beta_{n}}\frac{1-\cos\left(\beta_{n}h_{2}\right)}{h_{2}\sin^{2}\left(k\beta_{n}h_{1}\right)+k_{0}h_{1}\sin^{2}\left(\beta_{n}h_{2}\right)},$$

where $k=\sqrt{\frac{D_{2}}{D_{1}}}$ and $\beta_{n}$ are the positive roots of the equation:

(11) $$\cot\left(\beta h_{2}\right)+\frac{k_{0}}{k}\cot\left(k\beta h_{1}\right)=0.$$

Here, it was assumed that *kh*_1_/*h*_2_ is an irrational fraction [12].

If a welded joint is homogeneous (*k*_0_ = 1, *D*_1_ = *D*_2_ = *D*), the solution of the problem takes a simple form:

(12) $$C\left(x,t\right)=\overset{\infty }{\underset{n=-\infty }{\sum }}[C_{01}\mathrm{erf}\left(\frac{x-2nh}{\sqrt{4Dt}}\right)-C_{02}\mathrm{erf}\left(\frac{x+\left(2n-1\right)h}{\sqrt{4Dt}}\right)\;-\frac{1}{2}\;\underset{m=-1,1}{\sum }\left\{C_{01}\mathrm{erf}\left(\frac{x-2nh+mh_{1}}{\sqrt{4Dt}}\right)-C_{02}\mathrm{erf}\left(\frac{x+\left(2n-1\right)h+mh_{2}}{\sqrt{4Dt}}\right)\right\}],$$

where $\mathrm{erf}\left(u\right)=\frac{2}{\sqrt{\pi }}\int_{0}^{u}e^{-u^{2}}\;du$.

If the lower surface of the plate does not affect the hydrogen diffusion, that is, the distance between the weld interface and the lower surface is great enough (theoretically *h*_2_→∞), then the hydrogen concentration distribution in such an inhomogeneous solid is described by the following equations [10]:

(13) $$C_{1}\left(x,t\right)=C_{01}-C_{01}\mathrm{erfc}\left(\frac{x}{\sqrt{4D_{1}t}}\right)+C_{01}\overset{\infty }{\underset{n=1}{\sum }}B^{n}\left[\mathrm{erfc}\left(\frac{2nh_{1}-x}{\sqrt{4D_{1}t}}\right)-\mathrm{erfc}\left(\frac{2nh_{1}+x}{\sqrt{4D_{1}t}}\right)\right]\;-k\frac{C_{01}-k_{0}C_{02}}{k+k_{0}}\overset{\infty }{\underset{n=1}{\sum }}B^{n-1}\left[\mathrm{erfc}\left(\frac{\left(2n-1\right)h_{1}-x}{\sqrt{4D_{1}t}}\right)-\mathrm{erfc}\left(\frac{\left(2n-1\right)h_{1}+x}{\sqrt{4D_{1}t}}\right)\right],$$

(14) $$C_{2}\left(x,t\right)=C_{02}+\frac{k_{0}}{k+k_{0}}\{\left(\frac{C_{01}}{k_{0}}-C_{02}\right)\mathrm{erfc}\left(\frac{x-h_{1}}{\sqrt{4D_{2}t}}\right)\;-\frac{2C_{01}}{k_{0}}\overset{\infty }{\underset{n=1}{\sum }}B^{n-1}\mathrm{erfc}\left(\frac{x-h_{1}+\left(2n-1\right)h_{1}\sqrt{D_{2}/D_{1}}}{\sqrt{4D_{2}t}}\right)\;+\frac{2k}{k+k_{0}}\left(\frac{C_{01}}{k_{0}}-C_{02}\right)\overset{\infty }{\underset{n=1}{\sum }}B^{n-1}\mathrm{erfc}\left(\frac{x-h_{1}+2nh_{1}\sqrt{D_{2}/D_{1}}}{\sqrt{4D_{2}t}}\right)\},$$

where $B=\frac{k-k_{0}}{k+k_{0}},\;\mathrm{erfc}\left(u\right)=1-\mathrm{erf}\left(u\right).$

The corresponding computer programs have been developed.

## 4. Results and Discussion

Let us consider hydrogen diffusion in a welded joint combining different materials conventionally named ferrite *F* (low-carbon low-alloy steel), martensite *M* (alloyed steel) and austenite *A* (high-alloy steel). Let the diffusion coefficients at a temperature of 293 K be 1.50 × 10^−9^ m^2^⋅s^−1^ for *F*, 1.58 × 10^−10^ m^2^⋅s^−1^ for *M*, and 3.98 × 10^−15^ m^2^⋅s^−1^ for *A* and the hydrogen solubility in γ-Fe (*A*) relative to α-Fe (*F* and *M*) is *k*_0_ = 4013. The *D* and *k*_0_ values correspond to iron, 2.25Cr-1Mo steel, and 347 stainless steel [13]. Hence, the diffusion coefficient in a quenched steel *M* is one order of magnitude less than that in a non-quenched steel *F* and five orders of magnitude greater than that in austenitic steel *A*. The weld thickness is *h*_1_ = 4 mm and the joint thickness is *h* = 10 mm (Figure 1a). Let the initial hydrogen concentration be *C*_01_ in the WM and *C*_02_ = 0.1 *C*_01_ in the BM, unless otherwise specified.

**Figure 2:**
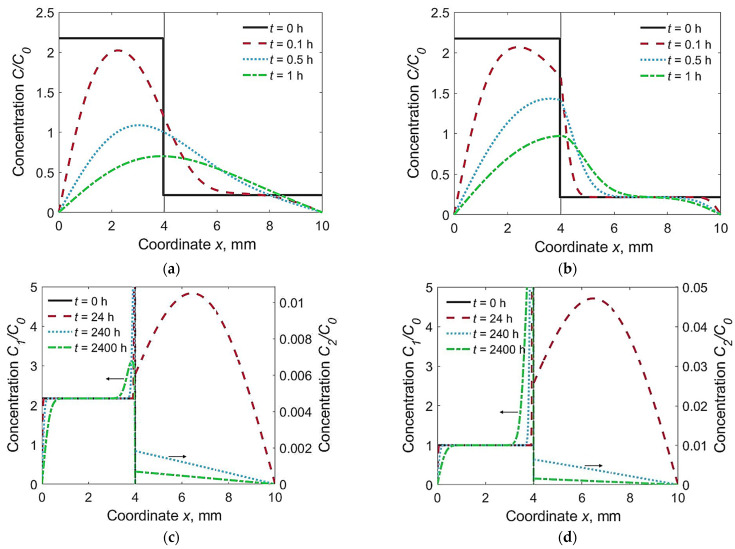
Distribution of relative hydrogen concentration in welded joints at different time *t*: (**a**) *F* + *F*, *C*_02_/*C*_01_ = 0.1; (**b**) *F* + *M*, *C*_02_/*C*_01_ = 0.1; (**c**) *A* + *M*, *C*_02_/*C*_01_ = 0.1; (**d**) *A* + *M*, *C*_02_/*C*_01_ = 1.

**Figure 3:**
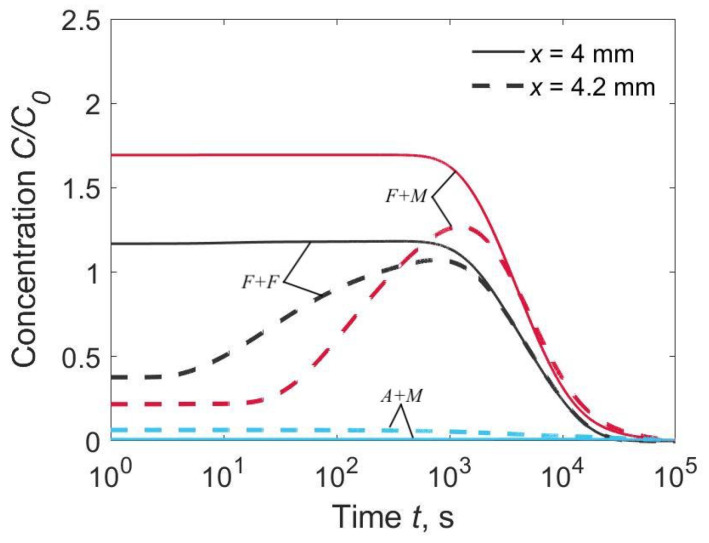
Time dependence of hydrogen concentration in the HAZ at the weld interface (*x* = 4.0 mm) and at 0.2 mm from the weld interface (*x* = 4.2 mm), *C*_02_/C_01_ = 0.1.

Three characteristic combinations of materials were under consideration: *F* + *F* (*D*_1_ = *D*_2_, *k*_0_ = 1), *F* + *M* (*D*_1_ > *D*_2_, *k*_0_ = 1) and *A* + *M* (*D*_1_ < *D*_2_, *k*_0_ = 4013). The first combination corresponded to a homogeneous joint with a WM chemistry that closely matches the BM. The second combination corresponded to the welding of alloyed steels with low-carbon non-alloyed welding consumables. The third combination corresponded to the welding of low-carbon alloyed steels with austenitic welding consumables. We determined the time-dependent hydrogen diffusion from the distribution of hydrogen over thickness (Figure 2) and the local concentration at the weld interface (*x* = *h*_1_) and at the HAZ (*x* = *h*_1_ + 0.2 mm, Figure 3).

To compare different solutions, we normalized the results to the average initial hydrogen concentration in the joint *C*_0_, *C*_0_ = (*h*_1_*C*_01_ + *h*_2_*C*_02_)/*h*.

In the homogeneous joint *F* + *F*, hydrogen flux was directed from the WM to the HAZ. Concentration of hydrogen was maximum at the weld interface, *C*_2max_(*h*_1_) = 0.5(*C*_01_ + *C*_02_) ≈ 1.20 *C*_0_ (Figure 2a and Figure 3). The calculated hydrogen distributions across the plate thickness resembled the experimental curves [9].

In the welded joint of the *F* + *M* type (*F* for WM, *M* for BM), the hydrogen concentration in the HAZ reached 170% of the average initial concentration (*C*_2max_ ≈ 1.70 *C*_0_) due to intensive inflow from the WM and low discharge into the BM (Figure 2b and Figure 3). Long dwell time of the HAZ under high hydrogen concentration is a contributing factor to cold cracking.

Hydrogen concentration in the HAZ of the *A* + *M* type joint was relatively low; it decreased from an initial value down to 0.011 *C*_0_ (Figure 2c and Figure 3). Note that the *C*_2_/*C*_0_ scale was expanded. In other words, the austenitic WM sucks off hydrogen due to its relatively high solubility, even though the initial concentration in the BM is less than that in the WM (Figure 2c). A dramatic increase in the hydrogen concentration in the austenitic WM near the weld interface was experimentally observed [9]. If the initial concentration in the BM was as high as in the WM (*C*_02_ = *C*_01_), it dropped to 0.048 *C*_0_ (Figure 2d). Later, the hydrogen was released slowly from the WM and diffuses fast in the BM. Hence if hydrogen concentration in the HAZ is considered, the effect of the utilization of austenitic consumables is equivalent to a dramatic reduction in the initial hydrogen content in non-austenitic consumables.

Comparative analysis of Equations (9)–(14) proved that under the assumptions made, the peak concentration at the weld interface *C*_2max_(*h*_1_) depends only slightly on the plate thickness [10,11]. It follows from Equation (14):

(15) $$C_{2\max}\left(h_{1}\right)=\frac{C_{01}+C_{02}\sqrt{\frac{D_{2}}{D_{1}}}}{k_{0}+\sqrt{\frac{D_{2}}{D_{1}}}}.$$

**Figure 4:**
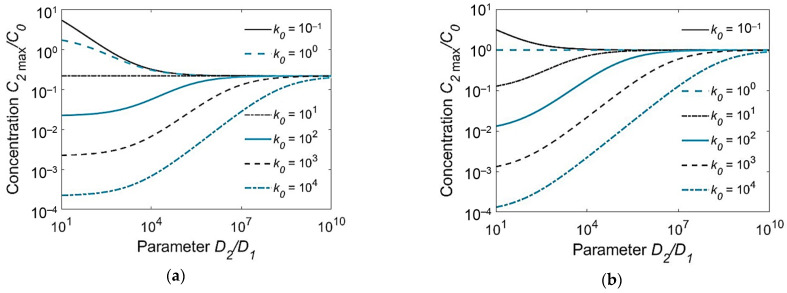
Peak hydrogen concentration in the HAZ at the weld interface (*C*_2max_(*h*_1_))/*C*_0_ as a function of the ratio *D*_2_/*D*_1_ of the diffusion coefficients and the partition coefficient *k*_0_: (**a**) *C*_02_/*C*_01_ = 0.1; (**b**) *C*_02_/*C*_01_ = 1.

It can be seen from Equation (15) that the peak concentration in the HAZ varies in direct proportion to the initial concentration in the WM *C*_01_ and in the BM *C*, and in inverse proportion to the partition coefficient *k*0 and the square root of the diffusion coefficient ratio of the BM and the WM *D*_2_/*D*_1_. This dependence is shown over a wide range of parameters in Figure 4. Thus, to minimize the hydrogen concentration near the weld interface and consequently hydrogen-induced cracking, welding consumables need to be chosen that have a lower diffusion coefficient (*D*_1_) and a greater solubility (partition coefficient *k*_0_) than the BM. The application of austenitic welding consumables with a low hydrogen content (*C*_01_) for the welding of alloyed steels (*D*_2_ ≫ *D*_1_, *k*_0_ ≫ 1) is an ideal case.

In practice, the behavior of hydrogen in a welded joint is commonly assessed based on evolution from the joint surface. Based on Equations (9)–(14), the density of hydrogen flux from a unit area of the surfaces *x* = 0 and *x* = *h* can be calculated as:

(16) $$J_{1}\left(0,t\right)=D_{1}{\left|\frac{\partial C_{1}\left(x,t\right)}{\partial x}\right|}_{x=0},$$

(17) $$J_{2}\left(h,t\right)=D_{2}{\left|\frac{\partial C_{2}\left(x,t\right)}{\partial x}\right|}_{x=h},$$

(18) $$J\left(t\right)=J_{1}\left(0,t\right)+J_{2}\left(h,t\right).$$

**Figure 5:**
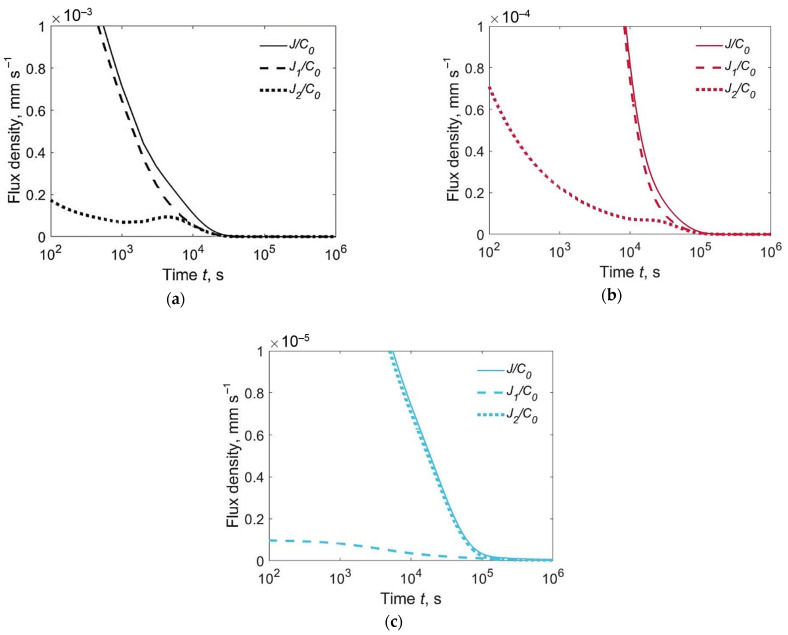
Relative flux density of hydrogen evolution from the upper surface *J*_1_/*C*_0_, lower surface *J*_2_/*C*_0_ and both surfaces *J*/*C*_0_ for welded joints of different types, C_02_/*C*_01_ = 0.1: (**a**) *F* + *F*; (**b**) *F* + *M*; (**c**) *A* + *M*.

Figure 5 shows the relative fluxes *J*_1_(0,*t*)/*C*_0_, *J*_2_(*h*,*t*)/*C*_0_ and *J*(*t*)/*C*_0_. The flux of effused hydrogen is usually the maximum at the weld metal surface (curves *J*_1_(0,*t*)/*C*_0_, Figure 5a,b), but this is not valid for an inhomogeneous joint with an austenitic weld metal and martensitic base metal (Figure 5c). In the latter case, the diffusion coefficient in the WM is several orders of magnitude lower than in the BM, due to which the hydrogen flux through the lower surface turns out to be noticeably greater than the flux through the weld surface, despite the significantly lower initial hydrogen concentration in the BM.

The flux density integrated over time from 0 to *t* is defined as the quantity of hydrogen evolved from a unit area of the corresponding surface by the time *t*:

(19) $$Q_{1}\left(0,t\right)=\overset{t}{\underset{0}{\int }}J_{1}\left(0,t\right)\;dt,$$

(20) $$Q_{2}\left(h,t\right)=\overset{t}{\underset{0}{\int }}J_{2}\left(h,t\right)\;dt,$$

(21) $$Q\left(t\right)=Q_{1}\left(0,t\right)+Q_{2}\left(h,t\right).$$

Here, *Q* is the total quantity of the evolved hydrogen. It can be computed alternatively as:

(22) $$Q\left(t\right)=Q_{0}-\overset{h}{\underset{0}{\int }}C\left(x,t\right)\;dx,$$

where *Q*_0_ is the initial quantity of hydrogen, *Q*_0_ = *C*_01_ *h*_1_ + *C*_02_ *h*_2_ = *C*_0_ *h*.

**Figure 6:**
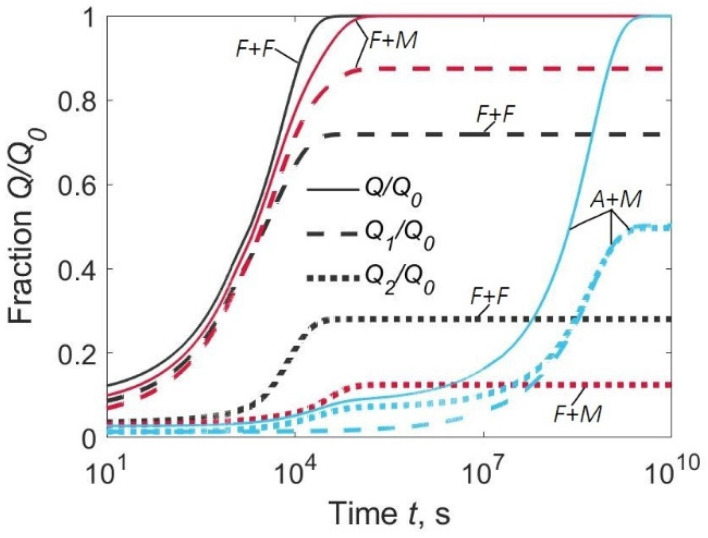
Fraction of hydrogen evolved from the upper surface (*Q*_1_/*Q*_0_), lower surface (*Q*_2_/*Q*_0_), and both surfaces (*Q*/*Q*_0_) for welded joints of three types: *F* + *F*, *F* + *M*, and *A* + *M*. *C*_02_/*C*_01_ = 0.1.

A significant proportion of hydrogen evolves from the lower surface if the diffusion coefficient of the BM is equal to or greater than that of the WM (curves *F* + *F* and *A* + *M*, Figure 6). The degassing times are about 10 h for an *F* + *F* type joint and several hundreds of hours for an *A* + *M* type joint (Figure 6).

It follows from a comparison of Figure 3 with Figure 5 and Figure 6 where there is no direct relationship between hydrogen concentration in the HAZ on one hand, and flux and the quantity of effused hydrogen on the other. This means that it is unjustified to link the current concentration in the HAZ to the experimental degassing curves, however, the scale of all values is given by the initial hydrogen concentration.

It must be noted that hydrogen diffusion coefficients for steels with a body-centered lattice can differ by 3–4 orders of magnitude at room temperature [14,15,16]. Therefore, for other combinations of materials, the calculated results will be quantitatively different from the ones obtained in this work; however, the main conclusions of the qualitative analysis will remain valid.

The obtained solutions of the hydrogen diffusion problem can be used to calculate the time and temperature of the post-weld heat treatment to reduce the hydrogen concentration to a permissible level. If the average temperature of a weld is assumed to change slowly, then the diffusion processes can be evaluated based on the concept of equivalent time [17].

It should be emphasized that the results of this study were obtained based on the exact solution of a mass transfer problem, corresponding to a significantly simplified physical model. For a more exact and detailed analysis of the diffusion processes, a more adequate physical model is required that takes into consideration anisothermal welding conditions, thermodiffusion (Soret effect), residual stresses, traps, and other factors. Under such approach, however, the problem of hydrogen diffusion can only be solved by using numerical methods [2,3,5,7,8].

## 5. Conclusions

(1) The obtained functional-analytical solutions make it possible to analyze hydrogen diffusion in inhomogeneous butt-welded joints considering the weld dimensions, initial hydrogen distribution, diffusion coefficients, and solubilities.
(2) The peak hydrogen concentration in the HAZ of inhomogeneous joints varies in direct proportion to the initial hydrogen concentration in the WM and in inverse proportion to the ratio of hydrogen solubilities in the WM and BM. It is nonlinear in the diffusion coefficient ratio of the BM and WM.
(3) In the welding of martensitic steel with ferritic welding consumables, the peak hydrogen concentration in the HAZ can exceed 170% of the average initial concentration in the joint, thus contributing to the susceptibility to cold cracking.
(4) Application of austenitic consumables leads to aa dramatic reduction in the hydrogen concentration in the HAZ in comparison with non-austenitic consumables.
(5) The time-dependent effusion of hydrogen depends on the inhomogeneity of the welded joint with regard to the solubility and diffusion coefficient. There is no direct relationship between the hydrogen concentration in the HAZ and the flux of effused hydrogen.

## Data Availability

Data sharing is not applicable.
